# A Novel Oncolytic Herpes Simplex Virus Type 2 Has Potent Anti-Tumor Activity

**DOI:** 10.1371/journal.pone.0093103

**Published:** 2014-03-26

**Authors:** Qian Zhao, Wen Zhang, Zhifeng Ning, Xiufen Zhuang, Haizhen Lu, Jing Liang, Jie Li, Yu Zhang, Ying Dong, Youhui Zhang, Shuren Zhang, Shangmei Liu, Binlei Liu

**Affiliations:** 1 Department of Pathology, Cancer Institute & Hospital, Chinese Academy of Medical Sciences & Peking Union Medical College, Beijing, China; 2 Department of Immunology, Cancer Institute & Hospital, Chinese Academy of Medical Sciences & Peking Union Medical College, Beijing, China; 3 School of Pharmacology, Hubei University of Science and Technology, Xianning, Hubei, China; Cincinnati Childrens Hospital Medical Center, United States of America

## Abstract

Oncolytic viruses are promising treatments for many kinds of solid tumors. In this study, we constructed a novel oncolytic herpes simplex virus type 2: oHSV2. We investigated the cytopathic effects of oHSV2 in vitro and tested its antitumor efficacy in a 4T1 breast cancer model. We compared its effect on the cell cycle and its immunologic impact with the traditional chemotherapeutic agent doxorubicin. In vitro data showed that oHSV2 infected most of the human and murine tumor cell lines and was highly oncolytic. oHSV2 infected and killed 4T1 tumor cells independent of their cell cycle phase, whereas doxorubicin mainly blocked cells that were in S and G2/M phase. In vivo study showed that both oHSV2 and doxorubicin had an antitumor effect, though the former was less toxic. oHSV2 treatment alone not only slowed down the growth of tumors without causing weight loss but also induced an elevation of NK cells and mild decrease of Tregs in spleen. In addition, combination therapy of doxorubicin followed by oHSV2 increased survival with weight loss than oHSV2 alone. The data showed that the oncolytic activity of oHSV2 was similar to oHSV1 in cell lines examined and in vivo. Therefore, we concluded that our virus is a safe and effective therapeutic agent for 4T1 breast cancer and that the sequential use of doxorubicin followed by oHSV2 could improve antitumor activity without enhancing doxorubicin’s toxicity.

## Introduction

Viruses are a promising treatment for solid tumors that act by selectively infecting and replicating in tumor cells, promoting cell lysis and producing progeny that can spread to other tumor cells [1]. Oncolytic viruses can also induce a specific antitumor immune response by releasing tumor-specific antigens that are recognized by CD8^+^ cytotoxic T lymphocytes (CTL) [2].

Many viruses have been employed as oncolytic agents, including adenovirus, reovirus and herpes simplex virus [2], [3], [4]. Oncolytic herpes simplex virus (oHSV) is usually constructed by deleting ICP34.5, a neurovirulence gene that restricts oHSV replication to tumor cells [5]. oHSV can effectively deliver various transgenes to assist in the treatment of tumors [6], [7]. Herpes simplex virus type 1 (HSV-1) has been widely used in past experimental and clinical studies [8], [9] due in part to its ability to selectively lyse tumor cells [9]. It was reported that an HSV-1-based oncolytic virus led to a reduction in tumor volume in both primary and metastatic tumors without causing weight loss in experimental murine 4T1 breast cancer models [10]. In another study, oncolytic HSV-1 expressing GM-CSF enhanced the tumor-specific immune response and reduced negative immunomodulatory cells (such as Treg and Ts cells) when injected into tumors [11]. In one study, almost all the primary human mammary carcinoma cells derived from fresh specimens were infected by oHSV-1 [1]. Moreover, oncolytic HSV-1 selectively infects breast cancer cells cocultured with bone marrow cells but does not infect the bone marrow cells or influence their hematopoietic function [1].

There are some indications that HSV-2 may be better for oncolysis than HSV-1. A domain of HSV-2 can activate the RAS/MEK/MAPK pathway and improve the efficiency of virus reproduction [12]. HSV-2 can selectively infect tumor cells and form syncytia, resulting in a better antitumor immune response than HSV-1 [12], [13]. Compared with an HSV-1 oncolytic virus, the HSV-2 oncolytic virus FusOn-H2 was more effective in killing MDA-MB-435 human breast cancer cells at a lower multiplicity of infection (MOI), resulting in more tumor-free mice [12]. Dying syncytia released more syncytiosomes, which allow dendritic cells (DCs) to work more effectively and result in more powerful antigen cross-presentation [14]. ICP_10_PK-deleted oHSV-2 virus ΔPK caused cancer cell death through both direct oncolytic function and induction of programmed cell death pathways [15]. Furthermore, an oHSV-2 virus was more effective in reducing metastasis in the abdominal cavity than an HSV-1 virus [16].

These studies prompted us to construct a novel HSV-2 oncolytic virus in which both ICP34.5 and ICP47 gene was deleted. The resulting virus, oHSV2, was evaluated in the 4T1 model to investigate its therapeutic potential in breast cancer and its induction of an immune response. Given that the chemotherapeutic drug doxycycline improves the intratumoral (i.t.) spread and apoptotic and necrotic activity of oncolytic HSV [17], we chose to test the antitumor effect of our virus in combination with a chemoagent. Doxorubicin (DOX) is an effective and widely-used chemotherapy drug for breast cancer treatment [18] that leads to apoptosis [19]. Thus, it was chosen to test our hypothesis that combined treatment of oHSV2 and DOX increases oncolytic activity in 4T1 tumor models.

## Materials and Methods

### Cells and virus

The highly metastatic, nonimmunogenic breast tumor cell line 4T1 derived from Balb/c mice was purchased from ATCC. The 4T1 cells were cultured in DMEM/F12 supplemented with 10% FBS and gentamycin and incubated at 37°C in a humidified atmosphere of 5% CO_2_. The other tumor cell lines used in this study, including human gastric cancer cell line BGC823, human colorectal cancercell line HT29, human renal cell carcinoma cell line Krause, human breast tumor cell line T47D, human osteosarcoma cell line U2OS, human nasopharyngeal carcinoma cell line CNE2Z, human hepatoma cell line HuH7, human lung cancer cell line PG, human prostate cancer cell line TSU, mouse glioma cell line GL261, mouse ovarian cell line TC-1, and mouse melanoma cell lines B16F10 and B16R[20], were from Cell Resource Center (IBMS, CAMS/PUMC) and cultured in DMEM/F12 supplemented with 10% FBS. HG52, an HSV2 strain, was obtained from British Health Protection Agency Culture Collection (HPA, Salisbury, United Kingdom).

### Mice and drugs

Six-week-old female Balb/c mice (Animal Center of the Chinese Academy of Medical Sciences, Beijing, China) were kept under specific pathogen-free conditions. Mice were treated according to the National Institutes of Health guidelines. The protocol was approved by the Committee on the Ethics of Animal Experiments of the Chinese Academy of Medical Sciences & Peking Union Medical College.

DOX was purchased from Shenzhen Main Luck Pharmaceuticals Inc. and diluted to 1 mg/ml in DMEM/F12 before use.

### Plasmid construction

A number of plasmids were constructed for the deletion of ICP47 and ICP34.5 genes from the HSV2 genome (HG52 strain). To delete ICP47, we constructed a plasmid that contains the up-stream (US) and down-stream (DS) flanking regions (FLRs) of ICP47 amplified by PCR. First, the US and DS FLRs were amplified with the primer pairs ICP47USf/ICP47USr and ICP47DSf/ICP47DSr, respectively (Table 1). Next, these two fragments were sequentially cloned into pSP73 to create pdICP47H2. The CMV-GFP-BGHpA fragment was digested from pcDNA3CMFGFP using Nru I/Pvu II and inserted into the pdICP47H2 EcoRV site to generate pdICP47H2GFP.

**Table1:** The primers used for the construction of pH2d34.5 and pdICP47H2 are shown, and the genome coordinates for the genomic sequences are indicated.

Primer name	Sequence
ICP34.5USf	AAAT***CAGCTG*** ^124356^CGGTGAAGGTCGTCGTCAGAG^124376^
ICP34.5USr	AAAT***TCTAGA*** ^125661^GCCGGCTTCCCGGTATGGTAA^125641^
ICP34.5DSf	AAAT***GATATC*** ^126943^CAGCCCGGGCCGTGTTGCGGG^126963^
ICP34.5DSr	AAAT***AGATCT*** ^12764^°CTCTGACCTGAGTGCAGGTTA^127620^
ICP47USf	^146554^AGAGTCACGACGCATTTGCCC^146574^
ICP47USr	^147775^ATACGATCTCGTCGACCGGGG^147755^
ICP47DSf	^148033^CATGGTGTCCCGTCCACGAAG^148053^
ICP47DSr	^149211^GGTTCGTGGTAATGAGATGCC^149191^

The restriction sites used for the construction of the shuttle plasmids are indicated by bold italics.

To amplify the up-stream (US) and down-stream (DS) flanking regions (FLRs) of ICP34.5, we used the primers listed in Table 1. First, the USFLR was amplified with ICP34.5USf and ICP34.5USr and inserted into the pSP72 PvuII/XbaI site to create pSP72H2d34.5US. Next, DSFLR was amplified with ICP34.5DSf and ICP34.5DSr and inserted into the pSP72H2d34.5US EcoRV/BglII site to generate pH2d34.5. Finally, the GFP expression cassette under the control of the CMV IE promoter was inserted into the pH2d34.5 EcoRV site to generate pH2d34.5-GFP. All plasmids were verified by sequencing.

### Virus construction

Oncolytic HSV2 is an attenuated oncolytic herpes simplex type 2 that was derived from the wild-type HSV-2 strain HG52. Oncolytic HSV2 was constructed using the following steps. First, HG52-d47 was constructed by deleting the ICP47 gene, which involved two homologous recombinations. The pdICP47H2-GFP plasmid (Table 1) replaced the primary ICP47 gene by co-transfection into BHK cells. The recombined vector (HG52-d47-GFP) was purified with four rounds of plaque assays using a fluorescent microscope. The GFP gene was removed by the pdICP47H2 plasmid (Table 1) using a similar procedure, which resulted in the HG52-d47 virus.

Next, both copies of the neurovirulence ICP34.5 gene in the HG52-d47 genome were deleted. The copies were replaced with the GFP-expressing cassette from the pH2dICP34.5-GFP plasmid (Table 1) by homologous recombination. They were purified as described above, resulting in oHSV2-GFP. The pH2dICP34.5 plasmid (Table 1) was used to replace the GFP gene in the oHSV2-GFP genome to obtain oHSV2.

oHSV1 with the same modifications as oHSV2 (ICP34.5 and ICP47 deleted) was constructed from oHSV1-GFP[21]. pdICP34.5 plasmid[21] was used to replace the GFP gene in the oHSV1-GFP genome to obtain oHSV1.

Virus stocks were prepared by infecting Vero cells (ATCC: CCL-81) with 0.01 plaque-forming units (pfu)/cell. Viruses were harvested 72 hours after infection, freeze-thawed once and purified by centrifugation at 1000 g for 10 mins to remove cell debris. This was followed by high-speed centrifugation at 17000 g for 1 h to pellet the virus. The virus pellet was dissolved in SFM, titrated, divided into aliquots, and stored at −80°C until use.

### In vitro characterization of oHSV2 in 4T1 cells

For in vitro phenotypic characterization of oHSV2 in 4T1 cells, the cells were seeded into 6-well plates at 2×10^5^/well. Twenty-four hours later, the cells were counted and either infected with viruses at 0.5, 1.0 and 5 MOI or left uninfected. The cells were cultured for another 24 hours before photomicrographs were taken.

### In vitro comparison of oHSV2 and oHSV1

Human and mouse cell lines, including 4T1, GL261, HT29, HuH-7, Krause, CNE2Z, T47D, TSU, PG, TC-1, B16F10, B16R [20] and U2OS, were placed in 12-well plates and infected with oHSV2 or its oHSV1 counterpart [21] at a MOI of 0.1 or 1 or left uninfected. Photomicrographs were taken 24 hours later.

### CCK8 cell viability assay

Cell viability was measured using a CCK-8 assay using Cell Counting kit-8 (DOJINDO, Japan). The cells were seeded at a concentration range from 2×10^4^ to 2×10^5^ cells/mL and were seeded in 96-well plates at 100 μL per well. Each sample was run in triplicate. To test the effect of multiplicity of infection (MOI) on cell viability, every cell line was infected 24 h later with oHSV1 or oHSV2. After culture for indicated time, the culture medium was removed from the plates, and 100 μl of a mixture containing 10% CCK8 was added and incubated for an additional 4 h. The plates were examined using a model 550 microplate reader (BIO-RAD, Japan) at 450 nm with a reference of 655 nm.

### Cell cycle analysis

The 4T1 cells were seeded in dishes (100-mm diameter), incubated overnight and counted. They received the following treatments: DOX at 0.5 μg/ml, 1 μg/ml or 2 μg/ml; oHSV2 at 0.1, 0.3 or 1 MOI or no treatment. The cells were incubated overnight, harvested and washed with PBS. After centrifugation, the cells were resuspended in PBS and their concentration was adjusted to 5×10^6^ cells/ml or 5×10^5^ cells/100 μl/test (n = 3). Each single-cell suspension was mixed with 1 ml 70% alcohol and stained at −20°C overnight. After centrifugation, the cells were resuspended with a cell cycle mix of PBS, PI (40 μg/ml), RNase (10 μg/ml) and Triton-100 (0.1%). After incubation for 30 minutes at 37°C, the cells were sorted with a flow cytometer (Becton Dickinson).

### Flow cytometry analysis

The cancer cells, including BGC823, HuH7, B16R, B16F10, and 4T-1, were seeded in dishes (100-mm diameter), incubated overnight. They received the following treatments: oHSV1 at 0.1, 0.3 MOI; oHSV2 at 0.1, 0.3 or no treatment. Cell apoptosis was measured using the Dead Cell Apoptosis Kit (Invitrogen, USA), according to the manufacturer’s instructions.

### Animal experiment

The 4T1 cells (5×10^4^) were suspended in 0.05 ml of DMEM/F12 SFM (Serum Free Medium) and subcutaneously (s.c.) injected into the right interscapular area of immune-competent female Balb/c mice (6 weeks old and16-17 g). The tumor size and body weight were measured 5 days after tumor inoculation when the tumors reached a palpable size. Tumor volumes were calculated with the following formula: volume = (length×width^2^)/2. The mice were separated into groups with an even distribution of tumor volumes (n = 12 per group). The groups were treated as follows: (a) DOX alone on days 0 and 3; (b) oHSV1 alone on days 0,2,4 and 6; (c) oHSV2 alone on days 0,2, 4 and 6; (d) DOX followed by oHSV2 (DOX/oHSV2), with DOX on days 0 and 3 and oHSV2 on days 5,7,9 and 11; (e) oHSV2 followed by DOX (oHSV2/DOX), with oHSV2 on days 0,2,4, and 6 and DOX on days 8 and 11; (f) concurrent oHSV2 and DOX (DOX+oHSV2) treatment with oHSV2 on days 0,2,4, and 6 and DOX on days 0 and 3 and (g) control treatment of DMEM/F12 SFM on days 0,2,4, and 6. For both oHSV1 and oHSV2, 3×10^6^ plaque forming units (pfu) were applied by direct intratumoral (i.t.) injection. DMEM/F12 SFM (200 μl) was injected i.t., and DOX was applied at a dose of 8 mg/kg by intra-peritoneum (i.p.) injection. The tumor size and body weight of each mouse were measured every 2 days following treatment and every 4 days since the 8th day of treatment, and the lifetime of the mice in each group was noted. In the experiments, tumor burden was determined by caliper measurement at indicated times after treatment, and mice were euthanized by cervical dislocation when tumor volumes reached 2000 mm^3^ or when animals showed distress, to avoid unnecessary suffering.

### Characterization of NK and Treg cells in spleens using flow cytometric analysis

The 4T1 cells (5×10^4^) were subcutaneously (s.c.) injected into the right interscapular area of female Balb/c mice (6 weeks old and16–17 g).The mice were treated with oHSV2 or DOX as animal experiment mentioned. And 7 days after the last treatment with oHSV2 or DOX, the spleens of mice (n = 3–4) were surgically removed and used for NK and Treg cell quantification. A single-cell suspension was prepared by filtration through a 400-gauge mesh. Lymphocytes from the spleens were isolated by centrifugation in a gradient lymphocyte isolation solution for mice (Tianjin Hao Yang Biological Manufacture Co., Ltd., China) at room temperature and washed twice with PBS. The cell suspensions were then stained at 4°C for 30 minutes using the following antibodies: FITC anti-mouse CD3, PE anti-mouse CD8b, Alexa Fluor®647 anti-mouse CD161, Alexa Fluor® 647 anti-mouse FOXP3 and their corresponding isotype control antibodies (all monoclonal antibodies were obtained from Biolegend). After washing with PBS, the cells were fixed with 10% formaldehyde. The NK and Treg cell frequency was determined using flow cytometry.

### Statistical analysis

All experiments were repeated triplicate unless otherwise stated. All quantitative data are reported as mean±SED. Statistical analysis was made for multiple comparisons using analysis of variance and Student’s t-test. P value < 0.05 was considered to be statistically significant.

## Results

### Construction of Oncolytic HSV2

**Figure 1:**
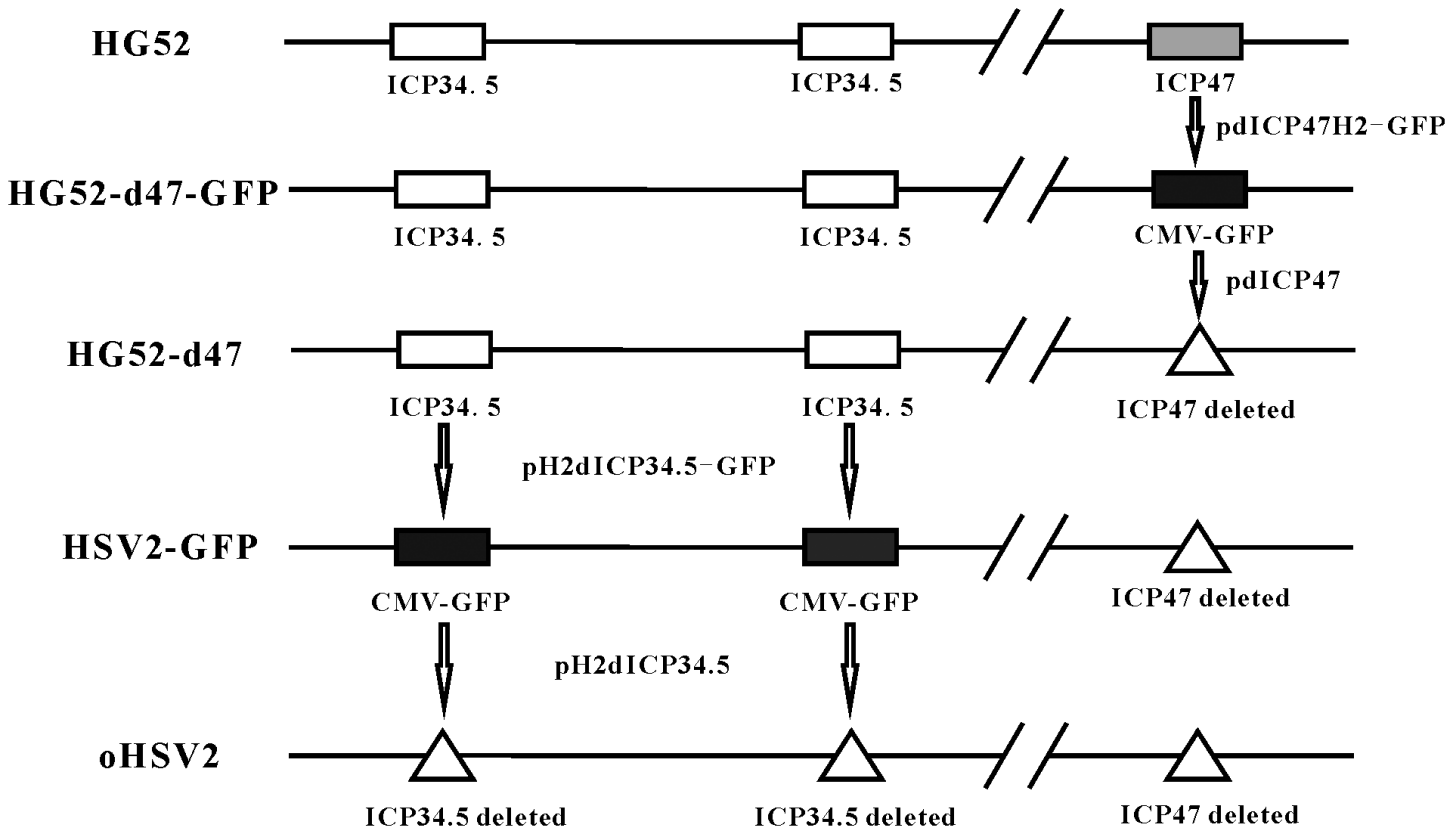
Schematic of the construction of oncolytic HSV2-GFP and HSV2. The two oncolytic HSV2 vectors were developed from the HG52 strain. Modifications include deletion of the ICP47 and ICP34.5 genes and insertion of a GFP expression cassette.

The construction of two recombinant oncolytic viruses, oHSV2-GFP and oHSV2, is depicted in Fig.1 (detailed procedures are described in the Materials and Methods). The genes encoding infected cell protein 34.5 (ICP34.5) and ICP47 were deleted. ICP34.5 is a neurovirulence gene that was removed to attenuate the toxicity of the virus and enhance its tumor selectivity. ICP34.5 resists interferon in normal cells; therefore, it is required for HSV to replicate in non-tumor cells. However, ICP34.5 is not necessary for viral replication in most tumor cells that have impaired interferon production or function. ICP47 was deleted because it can lower the expression of major histocompatibility complex (MHC) I in infected cells, which interferes with the presentation of tumor associated antigen (TAA). Also, the deletion of ICP47 results in the up-regulation of gene US11, which promotes virus oncolytic activity.

### oHSV2 possesses strong oncolytic activity that is equal to oHSV1 oncolytic activity in vitro

**Figure 2:**
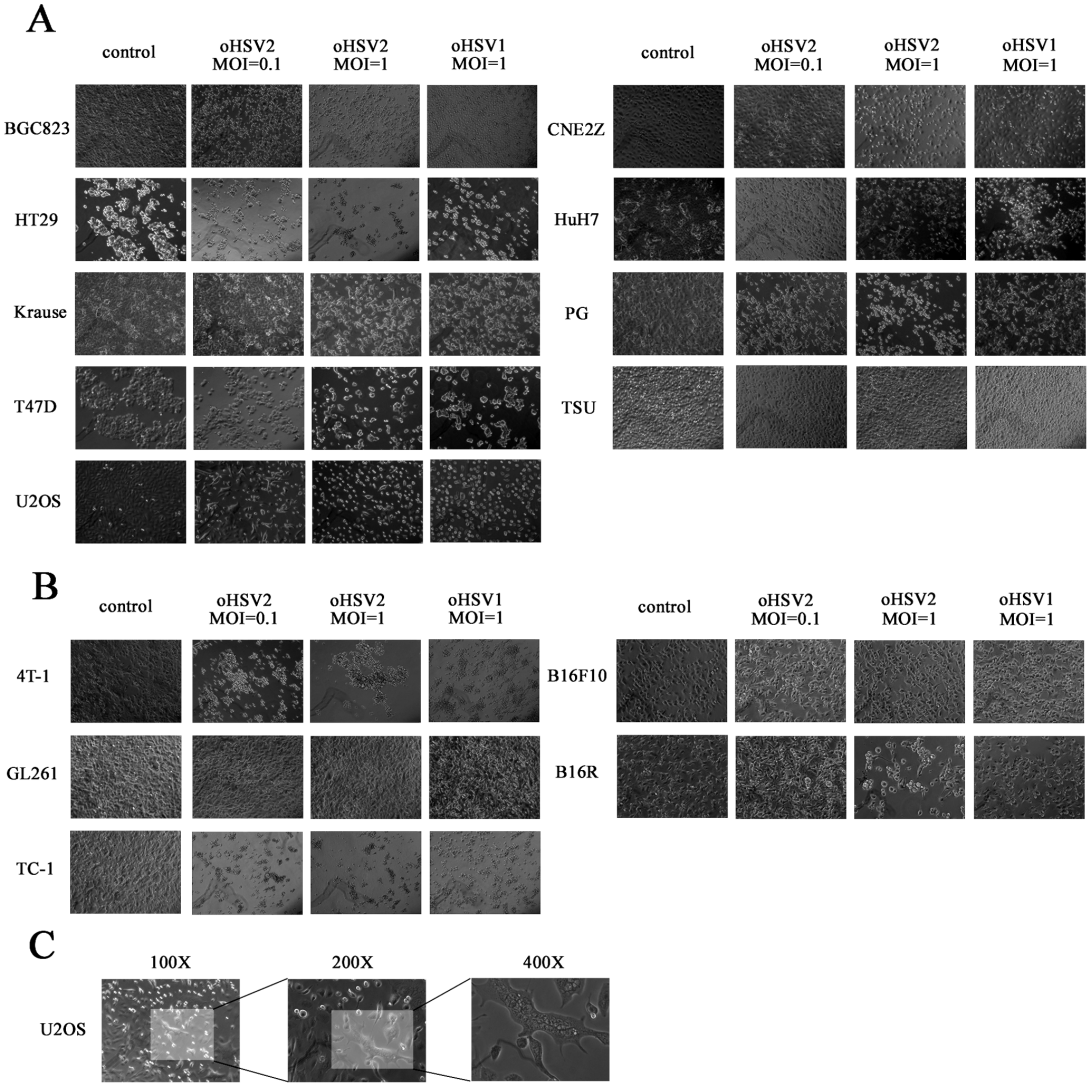
Oncolytic spectrum of oHSV2. **A**) oHSV2 and oHSV1 were used to infect human tumor cells, including BGC823, HT29, Krause, T47D, U2OS, CNE2Z, HuH7, PG and TSU, at the indicated MOIs and times. **B**) oHSV2 and oHSV1 were used to infect mouse tumor cells, including 4T1, GL261, TC-1, B16F10 and B16R, at the indicated MOIs and times. A and B were observed with an inverted phase contrast microscope at 100× objective magnification. **C**) U2OS cells were infected by oHSV2 at MOI = 1, and typical syncytia were observed at 100×, 200× and 400× objective magnification.

To investigate the oncolytic activity of oHSV2, its induction of a cytopathic effect (CPE) in vitro was compared with that of oHSV1. oHSV2 and oHSV1 with different MOIs were used to infect various human and mouse tumor cell lines. As shown in Fig.2, oHSV2 had an oncolytic effect on most of the human tumor cell lines, and the CPE of oHSV2 was equal to that of oHSV1 (Fig. 2 and Table 2). It is interesting that both oHSV2 and oHSV1 failed to induce CPE in the human prostatic carcinoma cell line Tsu (Fig. 2C). In addition, the CPE of oHSV2 was generally equal to the CPE of oHSV1 in mouse tumor cell lines that had HSV receptors. As shown in Fig. 2B, both oHSV2 and oHSV1 were able to induce CPE in B16R, which was stably transfected with an HSV receptor, herpes virus entry mediator (HVEM). However, they had little effect on wild-type B16F10. Additionally, oHSV2 but not oHSV1 caused the human osteogenic sarcoma cell line U2OS to form a typical syncytium, which was associated with strong induction of an antitumor immunological effect.

**Table 2:** Average CPE rates of cell lines. Cell lines were infected with oHSV1 or oHSV2 at MOI = 1 for 24 h.

Cell line	Average CPE rates (10 microscopic fields, 200×)		Total CPE/Total cells (200×)	
	oHSV2 (MOI = 1)	oHSV1 (MOI = 1)	oHSV2 (MOI = 1)	oHSV1 (MOI = 1)
BGC823	93.47±5.42%	75.61±7.9%	496/530	559/743
CNE2Z	39.47±7.79%	40.54±10.09%	267/686	271/672
HT-29	94.04±3.95%	83.18±3.26%	374/398	339/409
HuH7	66.03±9.74%	64.60±6.42%	352/534	338/522
Krause	27.81±7.43%	28.88±6.22%	181/654	194/656
PG	51.48±10.29%	52.56±8.31%	324/635	337/652
T47D	60.85±11.27%	71.66±9.13%	275/453	314/432
TSU	0%	0%	-	-
U2OS	60.33±5.59%	61.12±6.64%	251/415	275/452
4T1	45.34±4.66%	51.74±5.33%	305/674	345/668
GL261	0%	0%	-	-
TC-1	100%	100%	-	-
B16F10	0%	0%	-	-
B16R	68.00±7.58%	50.00±4.71%	462/677	355/712

10 fields of vision under the microscope were calculated and each CPE rate were calculated in the formula: CPE rate  =  number of CPE cells/number of total cells. The total CPE cells and total cells in the 10 microscopic fields were showed next to the percentages. The CPEs were observed with an inverted phase contrast microscope at 200× objective magnification.

**Figure 3:**
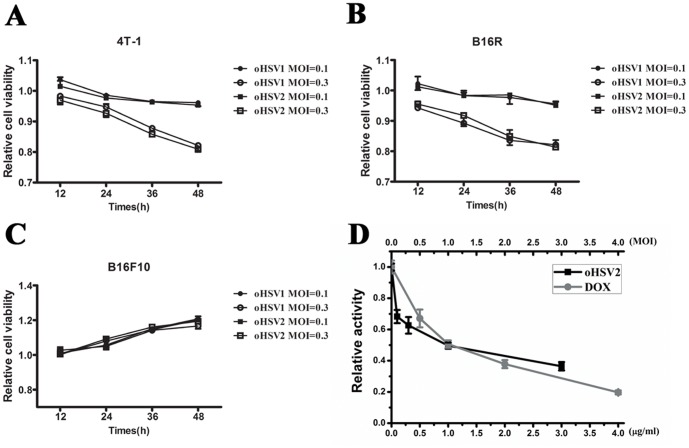
The cell viability of cancer cells was examined. **A**) The 4T-1 cells were treated with oHSV1 or oHSV2 at the indicated MOIs for the indicated times. **B**) The B16R cells were treated with oHSV1 or oHSV2 at the indicated MOIs for the indicated times. **C**) The B16F10 cells were treated with oHSV1 or oHSV2 at the indicated MOIs for the indicated times. **D**) The 4T-1 cells were treated with oHSV2 of different MOIs for 24h. DOX was used as a positive control. Each value represents the mean ± SED of three independent samples.

**Figure 4:**
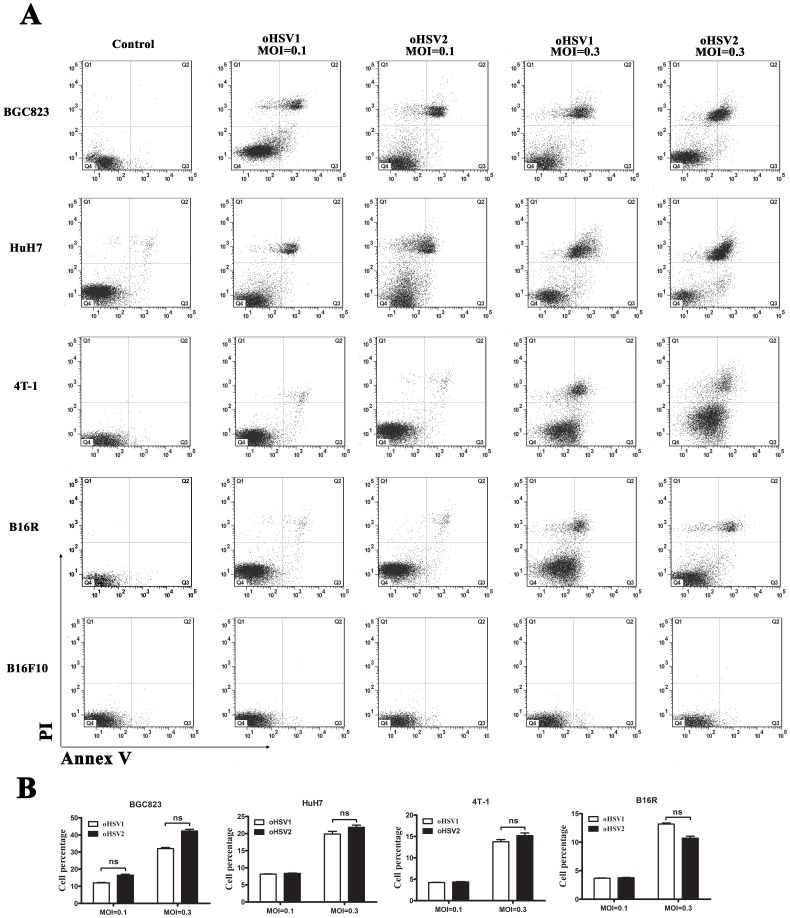
*In vitro* comparison of oHSV2 with oHSV1. Both oHSV1 and oHSV2 induces necrosis in cancer cells. A) Flow cytometry analysis of cancer cell lines after oHSV2 or oHSV1 infection at the indicated MOIs for 24 h. B) The necrosis rates of the cancer cell lines were measured after oHSV2 or oHSV1 infection. Each value represents the mean ± SED of three independent samples. ns, no significant differences.

We also investigated the oncolytic activity of oHSV2 at lower dose (MOI = 0.1 and 0.3) in comparison with oHSV1 by CCK8 assay. As shown in Fig. 3A and 3B, both oHSV2 and oHSV1 could decrease cell viability on 4T-1 and B16R after treatment with MOI = 0.3. However, they had little effect on wild-type B16F10 (Fig 3C). Additionally, the Annexin-V/PI assay showed that both oHSV2 and oHSV1 induced necrosis in tumor cells even in low dose, but not apoptosis (Fig 4A and 4B). It appeared that there was no significant difference between oHSV2 and oHSV1 in necrosis induction.

### oHSV2 has a cytotoxic effect on semi-permissive 4T1 tumor cells in vitro

To further examine the effect of oHSV2 on 4T1 cells in vitro, the cells were treated with different MOIs (MOI = 0.1, 0.3, 1, 3) for 24 hours and subsequently investigated for changes in cell viability using CCK8. We also applied different doses of DOX, a classic chemotherapy agent for human breast cancers, as a positive control. Both treatments exhibited a concentration-dependent reduction in tumor cell viability. As shown in Fig. 3D, the cells treated with 1 μg/ml DOX for 24h had cell viability of 50.64%. Viability decreased to 19.71% with 4 μg/ml DOX treatment. The viability of cells treated with oHSV2 also decreased, with 36.46% cell viability after treatment with MOI = 3.

### In vitro characterization of the effect of oHSV2 on the cell cycle of 4T1 tumor cells

**Figure 5:**
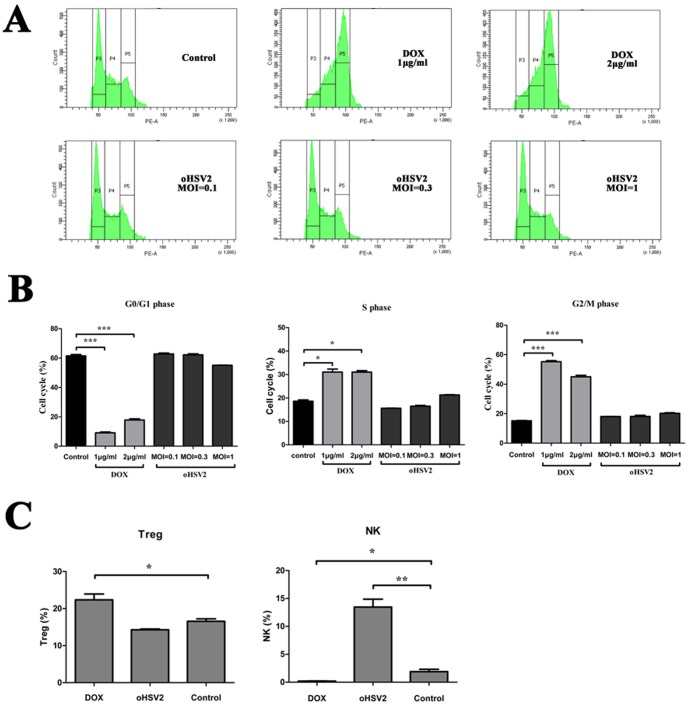
The oncolytic effect of oHSV2 on 4T1 tumor cells is independent of the cell cycle, but oHSV2 increases the NK ratio in vivo. A) An cell cycle assay as described in the Methods section. Representative images of flow cytometry from the different groups are depicted. B) 4T1 cells were treated with oHSV2 or different doses of DOX for 24h. Cell cycle specificity analysis was performed using flow cytometry. C) The percentage of NK and Treg cells after oHSV2 or DOX treatment was assayed. Statistical analysis was performed using an unpaired Student’s t test: *, p<0.05; **, p<0.01; and ***, p<0.001.

We next investigated whether oHSV2 treatment, like DOX, is sensitive with tumor cells in certain phase of the cell cycle. Cell cycle specificity analysis was performed using flow cytometry. As shown in Fig. 5A and 5B, treatment with DOX (2 μg/ml) had a marked effect on G2/M phase and S phase frequency (44.97% and 31.15%, respectively) compared with the control group (15.25% and 18.9%, respectively). The G0/G1 phase frequency of cells treated with 2 μg/ml DOX was significantly decreased to 17.35% (61.25% of control group). However, treatment with oHSV2 had no any effect on G2/M phase, S phase or G0/G1 phase compared with the control group. This implies that the oncolytic effect of oHSV2 on 4T1 tumor cells is independent of the cell cycle.

### oHSV2 treatment affects NK and Treg cells in murine spleen compared with DOX treatment

To explore whether oncolytic HSV2 or DOX treatment induces immunological changes in mice, we assessed the percentage of NK and Treg cells in the spleens of mice after each treatment by flow cytometric analysis. As shown in Fig. 5C, DOX treatment reduced the NK frequency in the spleens (0.20%) compared with the control group (3.75%). In contrast, oHSV2 treatment had a marked effect on NK frequency (13.64%) compared with the control group.

The Treg frequency in spleens treated with DOX was elevated (22%) compared with the control group (16.59%). However, oHSV2 treatment appeared to slightly reduce Treg levels compared with the control group.

### DOX coupled with HSV2 had an increased therapeutic effect in vivo

**Figure 6:**
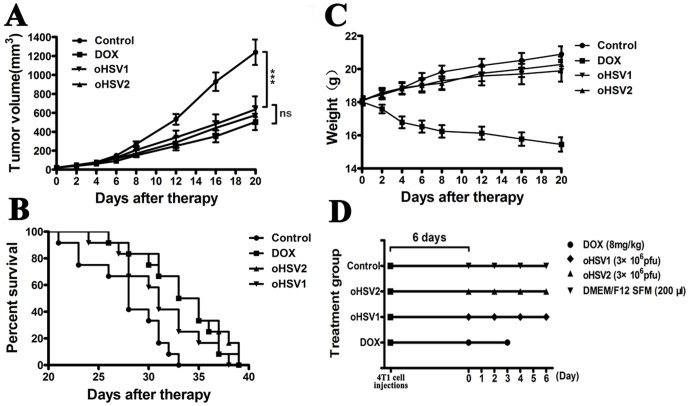
Anticancer effect of DOX, oHSV1 and oHSV2 in 4T1 breast tumors. The mice bearing 4T1 tumors were treated with DOX, oHSV1 or oHSV2 as described in the Materials and Methods section. **A**) The tumor volume was measured every 4 days following treatments. The data are presented as the mean ± SEM (n = 12), p<0.001. **B**) The median survival times for the 3 groups are illustrated in Kaplan–Meier survival curves (n = 12). Median survival: Control, 28 days; DOX, 34 days, p = 0.0034; oHSV2, 34 days, p = 0.0009; and oHSV1, 31 days, p = 0.0043. **C**) The weight of the mice was measured every 4 days following treatments. The data are presented as the mean ± SEM (n = 12). ***, p<0.001 and ns, no significant differences. D) Schematic of the experimental design. Each spot represents one treatment.

The results described above prompted examination of the anticancer effects of the different treatments in vivo. As shown in Fig. 6B, DOX, oHSV1 and oHSV2 each could prolonged the survival of mice relative to vehicle treatment (Median survival: 28 days for the control vs 34 days for DOX alone, 34 days for oHSV2 alone and 31 days for HSV1 alone; n = 12; p =  0.0034, 0.0433, and 0.0009 for DOX, oHSV1, and oHSV2, respectively, log-rank test). The group treated with DOX had a reduction in body weight (Fig. 6C), but there was no statistically significant change in body weight for the oHSV1, oHSV2 and control groups.

**Figure 7:**
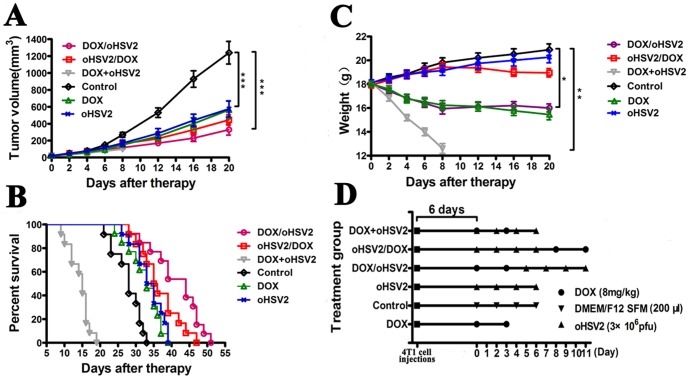
Anticancer effect of DOX coupled with oHSV2 in different treatment combinations in 4T1 breast tumors. Mice bearing 4T1 tumors were treated as described in the Materials and Methods section. And the Control, oHSV2 and DOX groups were the same in Fig 6 and 7. **A**) The tumor volume of mice was measured every 4 days following treatments. The data are presented as the mean ± SEM (n = 12). **B**) The median survival times for the 3 groups are illustrated in Kaplan–Meier survival curves (n = 12). Median survival: DOX/oHSV2, 44 days, p<0.001; oHSV2/DOX, 35 days, p<0.001; and DOX+oHSV2, 15 days. **C**) The weight of mice was measured every 4 days following treatments. The data are presented as the mean ± SEM (n = 12), *, p<0.05; **, p<0.01; and ***, p<0.001. D) Schematic of the experimental design. Each spot represents one treatment.

We also analyzed the anticancer effects of various combined treatments in vivo (Fig. 7). Mice treated with DOX followed by oHSV2 had a prolonged median survival time compared with mock-treated mice or oHSV2 group (28 days for the control group, 44 days for DOX/oHSV2 group and 34 days for oHSV2 group; n = 12; p<0.0001and p = 0.0021 for control and oHSV2, respectively, log-rank test). There was also significant difference between the median survival time of mice that received oHSV2 followed by DOX and mock-treated mice. However, concurrent treatment with oHSV2 and DOX led to a much shorter median survival time (15 days for DOX+oHSV2 group; n = 12). In addition, mice treated with DOX followed by oHSV2 experienced a significant reduction in tumor volume compared with the vehicle-treated control group. The group treated with oHSV2 followed by DOX did not lose weight relative to the mock-treated mice, whereas the group receiving concurrent DOX and oHSV2 treatment lost significant weight, indicating the importance of applying combination treatment in the appropriate sequence. It was also noted that mice in the group treated with DOX followed by oHSV2 were lighter in weight than the control group. Our results demonstrate that single treatment with either DOX or oHSV2 has anticancer effects but that combination treatment, especially DOX followed by oHSV2, demonstrates an increased anticancer efficacy without enhancing DOX toxicity in vivo.

## Discussion

At present, most cancers remain difficult to cure. Oncolytic virotherapy is a novel way to eradicate cancer cells that uses live viruses to selectively replicate in cancer cells and induce cell lyses with only minimal toxicity to normal tissues [22]. Various viruses are undergoing preclinical or clinical investigation, including vaccinia, adenovirus, herpes simplex virus, reovirus, and Newcastle disease virus [23]. Herpes simplex virus (HSV) is one of the most common oncolytic viruses and is reported to be an effective agent in melanoma phase 3 clinical trials [24]. A couple of reports compared the antitumor effects of oHSV1 and oHSV2 [25], [26]. However, direct comparison of the viruses is not appropriate because of significant differences in their modifications. In this study, we constructed oHSV1 and oHSV2 with identical modifications and compared their oncolytic activity. We have confirmed that both oHSV1 and oHSV2 have strong in vitro oncolytic activity in a series of human and murine cancer cell lines. The oncolytic effect of oHSV2 was equal to that of oHSV1 with the same MOI, and typical syncytia were observed in some cell lines infected by oHSV2. It is known that virus with higher particle to pfu ratio may induce more CPE in some cell types. In our study, the particle to pfu ratio is not known for both oHSV1 and oHSV2. However, both viruses were produced at the same time with the same conditions. Our data indicate that oHSV2 has potential for cancer therapy. So far, a number of oncolytic HSVs have shown potent oncolytic activity, and could induce strong immune responses against tumors in 4T-1 model [27],[28],[29]. The current study with the newly constructed oHSV2 further confirmed these findings.

DOX is commonly used in the treatment of a wide range of cancers, including hematological malignancies and many types of carcinoma [30]. It is also an anthracycline drug used for human breast cancer and was used in this study as a positive control. DOX is a cell cycle-dependent drug that interacts with DNA and inhibits macromolecular biosynthesis [31], [32]. We compared the effects of oHSV2 and DOX on 4T1 cells in vitro and showed that oHSV2 has cytotoxic effect at certain MOI. Although DOX has excellent anticancer activity in the clinic, it is associated with toxicity, including severe myelosuppression and dose-cumulative cardio-toxicity. Treatment of breast cancer with DOX does not eradicate cancer cells in certain phases of the cell cycle. For instance, we showed that only cells in G0/G1 phase are sensitive to DOX, which may result in the induction of drug resistance. However, effectiveness of oHSV2 was not cell cycle-dependent such that oHSV2 had an oncolytic effect on cancer cells in all cell cycle phases. Therefore, oHSV2 treatment can compensate for the deficiencies of DOX.

For our study, we used a mouse subcutaneous breast cancer model to evaluate the therapeutic effect of oHSV2.We examined the mouse spleen after different treatments and found that DOX treatment increased Treg cells and significantly decreased NK cells. To date, the effect of DOX on the anticancer immune response has been controversial [33], [34], [35]. Our data shows that DOX treatment may enhance negative regulation of the immune response. In contrast, oHSV2 treatment favorably enhanced the anticancer immune response by reducing Treg cells and increasing NK cells.

We previously reported that oncolytic HSV1 can reduce 4T1 tumor volume [21]. In the current study, we compared the effects of oHSV1 and oHSV2 on a mouse breast cancer model. As we expected, there was no difference between the median survival time of mice treated with oHSV2 and oHSV1.

Although our oHSV2 has a therapeutic effect on a 4T1 tumor model, it also has some limitations. These include the penetration of physical barriers such as the extracellular matrix, which restricts the initial distribution and subsequent spread of viruses in the tumor mass after intratumoral injection of the oncolytic virus. This limitation may be overcome by combining the virus with chemotherapies. Ideally, chemotherapy and oHSV2 with different mechanisms of action could improve antitumor activity. In our in vivo study, treatment with DOX followed by oHSV2 was significantly more beneficial than treatment with either agent alone. Although oHSV2 and DOX each have limitations, their combination may increase oncolytic efficacy and minimize toxic side effects.

In conclusion, this study showed that oHSV2 has a strong oncolytic effect on various human and mouse tumor cell lines. The overall oncolytic effect of oHSV2 was equal to that of oHSV1. In addition, treatment of 4T1 breast tumors with DOX chemotherapy followed by oHSV2 generated an enhanced anticancer effect in vivo than oHSV2 or DOX alone.
